# Stressors in hospitalized patients and their associations with mental health outcomes: testing perceived social support and spiritual well-being as moderators

**DOI:** 10.1186/s12888-023-04833-6

**Published:** 2023-05-09

**Authors:** Sarah Gerges, Rabih Hallit, Souheil Hallit

**Affiliations:** 1 School of Medicine and Medical Sciences, Holy Spirit University of Kaslik, P.O. Box 446, Jounieh, Lebanon; 2 Department of Infectious Disease, Notre Dame, Secours University Hospital Center, Street 93, Postal Code 3, Byblos, Lebanon; 3 Department of Infectious Disease, Bellevue Medical Center, Mansourieh, Lebanon; 4 Applied Science research Center, Applied Science private university, Amman, Jordan; 5 Research Department, Psychiatric Hospital of the Cross, Jal Eddib, Lebanon

**Keywords:** Hospital, Inpatients, Psychological distress, Stress, Depression, Anxiety, Psychological adaptation

## Abstract

**Background:**

Although hospitalization can be a burdensome experience for all patients, research into the sources of this distress and potential protective factors has so far been scattered, specifically among the broad hospitalized population across all disease types and inpatient units. The present study explores the frequency and nature of the foremost experienced hassles among a sample of Lebanese hospitalized patients, tracing their correlations with depression and anxiety while also investigating positive coping (i.e., perceived social support and spiritual well-being) as potential moderator of these relationships.

**Methods:**

A total of 452 Lebanese inpatients from all medical units filled a survey composed of a list of 38 stressors experienced during hospitalization and other measures assessing depression, anxiety, perceived social support, and spiritual well-being.

**Results:**

Pain was the most common stressor experienced by the patients (88.9%), followed by the feeling of being overwhelmed (80.3%). When conducting a factor analysis, 18 stressors loaded on 4 distinct factors, hence yielding 4 main stressor groups (i.e., Illness Apprehension, Hopelessness/Uselessness, Social Isolation, and Spiritual Concerns). The multivariable analysis showed that increased illness apprehension (Beta = 0.69) and hopelessness (Beta = 1.37), being married (Beta = 1.17) or divorced (Beta = 1.38) compared to single, being admitted in a two-bed room compared to one-bed (Beta = 1.59), higher financial burden (Beta = 0.24), and lower socio-economic status (Beta = 1.60) were significantly associated with higher anxiety. Additionally, increased hopelessness (Beta = 0.82) and being married (Beta = 0.79) compared to single were significantly associated with higher depression. However, among patients experiencing high levels of stressors, those with high spiritual well-being and perceived social support had lower depressive/anxiety symptoms.

**Conclusion:**

Our study characterized the principal stressors encountered during hospitalization, underscoring their associations with Lebanese inpatients’ mental health. On the other hand, as perceived social support and spiritual well-being acted as negative moderators of these associations, intervention programs aimed at enhancing such adaptive coping techniques are strongly called upon to palliate the psychological distress of patients in hospital settings.

## Introduction

Hospitalization is often accompanied by high levels of distress, which can engender considerable psychiatric comorbidities; worsen disease severity and patient disability; prolong hospital stays; and heighten hospital’s cost, burden, and rates of readmission [1–4]. Numerous investigations have indeed highlighted the intractable impact of hospitalization on the development of depressive and anxiety symptoms. For instance, a meta-analysis of 31 studies estimated that the prevalence of depression among general medical and surgical hospital inpatients ranges between 5% and 34%, with an average rate of 12% [5]. Likewise, another meta-analysis of 32 studies calculated prevalence estimates of 3%, 5%, 8%, and 28% for panic disorders, generalized anxiety disorders, anxiety disorders (all types), and anxiety symptoms among general hospital inpatients, respectively [6].

In return, it is noteworthy that positive psychology/psychiatry interventions (PPIs), a subset of psychosocial interventions, have shown promising benefits in alleviating such distress in medical patients [7]. Nonetheless, most of the previous analyses of depressive/anxiety symptoms and their predicting variables in the hospital context have focused on specific inpatients populations, namely cardiac/cardiovascular patients [8, 9], surgical patients [10–12], high-risk obstetrical patients [13], patients with Human Immunodeficiency Virus (HIV) [14], or patients with chronic diseases [15] such as diabetes mellitus [16] or malignancy [17, 18].

For example, within the Lebanese population, a previous study found high levels of depression, anxiety, and stress (21.3%, 61.3%, and 48.7%, respectively) among hospitalized patients with chronic illnesses, showing that avoidant coping styles, comorbidities, educational level, and female gender were significantly associated with higher ratings on psychological distress [15]. Another Lebanese study discovered a very high rate of anxiety (61.4%) among inpatients with substance use disorders, with better educated people experiencing less severe symptoms [19]. Furthermore, an investigation of suicidal risk among Lebanese psychiatric inpatients revealed that 37.5% tested positive for acute suicidal ideation; however, high spiritual well-being was significantly associated with lower suicidality in this specific inpatient population [20].

Nonetheless, to our best knowledge, only one precedent study has attempted to portray the sources of hospital discomfort and how they relate to anxiety among a heterogenous hospital-wide sample of inpatients from the United States [21]; nonetheless, it did neither address depressive symptoms nor explore factors that might have lessened the negative effects of hospital’s stressors on mental health. Consequently, owing to a scarcity of studies scrutinizing the reasons behind hospitalization-related depression and anxiety among the broad medical inpatient, it has been somewhat challenging to tailor treatment interventions and maximize their effectiveness among this general, though vulnerable, population [7].

Actually, bolstering positive mental states, cognitions, emotions, attitudes, and behaviors through a systematic execution of intentional exercises is what PPIs call for [22]; they require patients to conduct activities in their everyday life, which can be either clinician-led or self-guided, to self-build positive resources and happy experiences and so improve well-being. Thus, they do not just work towards assuaging psychological distress, which make them surpass traditional psychological therapeutic approaches (e.g., cognitive-behavioral therapy) [22]. As a result, PPIs can be widely applied in healthcare settings to aid in the prevention and treatment of depressive/anxiety symptoms and promote general well-being when dealing with diseases, as they are generally applicable to patients with and without psychopathology [7, 22–25]. Namely, thankfulness, seeking hope/faith and purpose in life (e.g., feeling good spiritually), fostering positive connections (e.g., feeling socially supported), kindness, or simply savoring are common themes pertaining to positive psychology [26]. However, while patients with somatic illnesses may strongly benefit from enhancements in their well-being through PPIs [27], to the best of our knowledge, no study has inquired about how positive psychology components, such as perceived social support and spiritual well-being, can modulate mental distress among hospitalized inpatients.

In sum, although hospitalization can be a burdensome experience for all patients, research into the sources of this distress and potential protective factors has so far been scattered, specifically among the general hospitalized population across all disease types and residing in all inpatient units. Moreover, treatment and support programs for depression and anxiety in the hospital have surprisingly been overlooked and/or underdeveloped, owing primarily to the paucity of research aimed at comprehending the emotional and environmental factors that trigger or assuage hospital-related psychological distress [7]. To this end, this works aims to (1) suggest a protective role for certain factors that would be negatively associated with mental distress among Lebanese inpatients, and thus, (2) preliminarily allude to potential therapeutic targets in the management of hospital-specific distress and associated mental disorders within Lebanese hospitals. Considering that this public health issue has received the least attention in developing countries, such contribution in Lebanon—a Middle-Eastern developing country—could enlighten the path towards implementing efficient PPIs and national mental health supporting policies for inpatients.

Therefore, in the present study, our objective was to explore the frequency and nature of the foremost experienced hassles among a sample of Lebanese hospitalized patients, tracing their correlations with depression and anxiety while also investigating positive coping/psychological adaptation (i.e., perceived social support and spiritual well-being) as potential moderator of these relationships. For exploratory purposes, we also evaluated the moderating effects of comorbidities and sociodemographic characteristics, such as age, gender, and the socio-economic status, in the relationships between stressors and mental health problems, relying on previous work highlighting the associations between these factors and mental health problems in hospitalized patients [15, 21, 28–30].

## Methods

### Study design

From November 2021 through January 2022, 452 Lebanese inpatients, distributed in all the inpatient medical units of a university hospital (i.e., The Centre Hospitalier Universitaire Notre Dame de Secours located in Byblos, Lebanon), took part in our cross-sectional survey. Eligibility was defined by being aged 18 years or over and able to read the Arabic language, whereas exclusion criteria were cognitive impairment, unconsciousness/unresponsiveness, and isolation. These exclusion criteria were assessed by looking at the medical records of the patients. At the start of each day within this period, the hospital records were reviewed, and all newly admitted eligible inpatients were approached and invited to participate. In total, 452 out of the 550 eligible hospitalized patients participated in the survey; the response rate was 82.2%, as 98 patients refused to participate. All the participants could access the Google Forms survey’s link via their smartphones, in order to answer the survey questions on their own. Participation was voluntary and anonymous. The study’s objectives were intelligibly stated in the introductory section of the survey, and patients had to read the instructions and consent to participate (by answering “yes”) before proceeding to the next sections.

### Minimal sample size calculation

The G*power 3.1.9.7 software (linear multiple regression: fixed model, R^2^ increase) [31] showed that a minimal sample of 395 inpatients was necessary to achieve satisfactory statistical power, when accepting a 5% risk of error, a 80% power, a small 2% effect size (f2) (as categorized by Cohen [32]), and 10 variables in the multivariable model.

### Questionnaire and variables

The questionnaires were administered in Arabic, Lebanon’s native language. The needed time for completion was 15 min. The first sections gathered data about socio-demographic characteristics, namely patient’s age, gender, marital status, educational level (i.e., primary, complementary, secondary, or university level), and experienced financial burden (rated from 1 to 10). The overall socioeconomic status of the participants was also appraised by the household crowding index, a measure that computes the ratio of the total number of people over the total number of rooms in the house except the bathrooms and kitchen. Higher ratios are indicative of lower socioeconomic households [33]. Questions tackling insurance type (i.e., private, national security, Lebanese army, and public health ministry), room type (i.e., one-bed or two-bed room), and general comorbidities (i.e., cardiovascular disease, hypertension, diabetes, chronic kidney disease, neurological disease, and psychiatric illness) were also included.

Additionally, based on a previous study scrutinizing stressors that correlated with anxiety symptoms among a general hospitalized population [21], we included **a list of 38 potential stressors** (displayed in Table 1). The endorsement of these stressors was categorized into Yes or No, after asking the patients if each particular stress factor was a source of burden during the current hospitalization (i.e., exclusively while in the hospital). The other parts of the survey comprised:

**Table 1:** Frequency of each Stressor (N = 452)

	N (%)
Pain	402 (88.9%)
Regret	184 (40.7%)
Feelings of low self-worth	218 (48.2%)
Feeling overwhelmed	363 (80.3%)
Feeling loss of control	226 (50.0%)
Feeling disconnected from family	130 (28.8%)
Not talking about what is going through	66 (14.6%)
Suffering is meaningless	134 (29.6%)
Loss of meaning/purpose in life	130 (28.8%)
Loneliness	116 (25.7%)
Hopelessness	118 (26.1%)
Frustration	186 (41.2%)
Guilt/shame	310 (68.6%)
Others judging me	269 (59.5%)
Need for forgiveness	307 (67.9%)
Fear of upcoming procedure	346 (76.5%)
Fear of death	139 (30.8%)
Fear of the unknown about diagnosis/treatment	304 (67.3%)
Worry about quality of life (QOL)	330 (73.0%)
Loss of bodily function	265 (58.6%)
Missing important events	180 (39.8%)
Disconnected from God	86 (19.0%)
God abandoned/punished me	82 (18.1%)
Questioning the faith	56 (12.4%)
Anger at God	44 (9.7%)
Concerns about afterlife	85 (18.8%)
Conflicts with hospital staff	84 (18.6%)
Guilt for being burden on family	344 (76.1%)
Inadequate support from family	100 (22.1%)
Other family member/friend ill	270 (59.7%)
Marital troubles	86 (19.0%)
Inability to sleep	237 (52.4%)
Difficulty accepting how I appear to others because of my illness	282 (62.4%)
Worry about care after discharge	159 (35.2%)
Financial burden	334 (73.9%)
Worry who will take care of the family	294 (65.0%)
Bother from noise/ light	162 (35.8%)
Environmental changes	137 (30.3%)

#### The patient health questionnaire (PHQ-9)

This brief 9-item tool is greatly efficacious to detect depression among clinical samples. Each item (e.g., “Little interest or pleasure in doing things” and “Feeling down, depressed, or hopeless”) is scored from 0 (i.e., “not at all”) to 3 (i.e., “nearly every day”), quantifying symptoms’ severity [34]. This scale was validated in Arabic among the Lebanese population [35]. (Cronbach’s alpha in this study = 0.88)

#### The lebanese anxiety scale (LAS-10)

This scale is a brief tool to screen for anxiety, composed of 10 items that were derived from the diagnostic criteria retained in the DSM-5, HAM-A, and STAI measures for anxiety. For instance, items include “I have an anxious mood (worries, anticipation of the worst, fearful anticipation, irritability)” and “I feel that difficulties are piling up so that I cannot overcome them”. Increased scores represent an escalation of anxiety symptoms. This scale was intentionally conceived and validated to depict anxiety among the Lebanese population [36, 37]. (Cronbach’s alpha in this study = 0.93)

#### The multidimensional scale of perceived social support (MSPSS)

This short 12-item instrument gauges self-perceptions of social support that comes from family, friends, and a significant person. It consists of three subscales, and each comprises 4 items. Items, which include “My family really tries to help me”, “I can count on my friends when things go wrong”, and “There is a special person in my life who cares about my feelings”, are rated from 1 (i.e., “strongly disagree”) to 7 (i.e., “strongly agree”) [38]. Higher scores denote a greater perceived social support. This measure was also validated in Lebanon [39]. (Cronbach’s alpha in this study = 0.96)

#### The functional assessment of chronic illness therapy 12-item spiritual well-being scale (FACIT-Sp-12)

This tool detains 3 subscales that assess faith, meaning of life, and peace (e.g., “I find comfort in my faith or spiritual beliefs”, “I feel a sense of purpose in my life”, and “I feel peaceful”). Response options range from “not at all” (scored as 0) to “very much” (scored as 4). The greater the total score, the higher the spiritual well-being [40]. (Cronbach’s alpha in this study = 0.89)

### Statistical analysis

To explore the factor structure of the 38 stressors, we computed a factor analysis using the FACTOR software [41, 42]. We verified all requirements related to item-communality [43], average item correlations, and item-total correlations [44]. The Kaiser-Meyer-Olkin (KMO) measure of sampling adequacy (which should ideally be ≥ 0.80) and Bartlett’s test of sphericity (which should be significant) ensured the adequacy of our sample [45]. The procedure for determining the number of factors to extract was parallel analysis (PA) [46], using the polychoric correlation matrix. We used the robust diagonally weight least squares to extract the factors and the weighted varimax for the rotation start. The rotation to achieve factor simplicity was done using the robust promin.

The SPSS software v.25 was used to conduct the remaining analyses. Anxiety and depression scores were normally distributed. The Student t and ANOVA tests were used to compared two and three or more means, respectively. Pearson test was used to correlate two scores. Two linear regressions were then conducted, taking the anxiety and depression scores as dependent variables, respectively. The absence of multicollinearity was verified through the calculation of the Variance Inflation Factor (VIF); VIF values < 5 indicate the absence of multicollinearity [47]. Moderation models were analyzed using the PROCESS macro v.3.4 model 1 [48]. Factors that showed a p < 0.25 were taken as independent variables in the model. Significance was set at p < 0.05.

## Results

### Sociodemographic and other characteristics of the participants

A total of 452 patients filled the survey (mean age: 47.60 years; 52.7% females). Details related to the marital status, education, insurance coverage, comorbidities, and other characteristics of the patients can be found in Table 2.

**Table 2:** Sociodemographic and Other Characteristics of the Patients (N = 452)

Variable	N (%)
**Gender**	
Male	214 (47.3%)
Female	238 (52.7%)
**Marital status**	
Single	111 (24.6%)
Married	232 (51.3%)
Divorced	72 (15.9%)
Widowed	37 (8.2%)
**Education**	
Primary	48 (10.6%)
Complementary	113 (25.0%)
Secondary	149 (33.0)
University	142 (31.4%)
**Private insurance**	
No	275 (60.8%)
Yes	177 (39.2%)
**National Social Security**	
No	258 (57.1%)
Yes	194 (42.9%)
**Lebanese Army**	
No	338 (74.8%)
Yes	114 (25.2%)
**Ministry of Public Health**	
No	409 (90.5%)
Yes	43 (9.5%)
**Number of Beds in the Room**	
One bed	65 (14.4%)
Two beds	387 (85.6%)
**Comorbidities**	
Cardiovascular	137 (30.3%)
Hypertension	146 (32.3%)
Diabetes	82 (18.1%)
Chronic kidney disease	44 (9.7%)
Neurological	103 (22.8%)
Psychiatric	29 (6.4%)
	**Mean ± SD**
Age (in years)	47.60 ± 16.88
Financial Burden	7.64 ± 2.04
Household Crowding Index	0.98 ± 0.39

### Prevalence of each stressor

Pain was the most common stressor experienced by the patients (88.9%), followed by the feeling of being overwhelmed (80.3%). The prevalence rates of the other stressors are summarized in Table 1.

### Factor analysis of the stressors

All stressors were entered in the factor analysis; all items with a factor loading > 0.4, a communality < 0.3 and a normed MSA > 0.5 were removed; 18 stressors remained at the end and loaded on four factors (F1: Illness Apprehension; F2: Hopelessness/Uselessness; F3: Social Abandonment; and F4: Spiritual Concerns), which explained 75.31% of the total variance. The Cronbach’s alpha of the four factors were excellent (Table 3).

**Table 3:** Factor (F) Analysis using the Principal Component Analysis of the Stressors using the Promax Rotation

	F1: Illness Apprehension	F2: Hopelessness /Uselessness	F3: Social Isolation	F4: Spiritual Concerns	Normed MSA
Regret (F2)	0.050	**0.460**	0.002	0.342	0.926
Feeling overwhelmed (F2)	-0.04	**0.751**	-0.084	-0.322	0.716
Feeling loss of control (F2)	-0.012	**0.879**	-0.047	0.038	0.906
Feeling disconnected from family (F3)	-0.078	0.372	**0.884**	-0.283	0.842
Suffering is meaningless (F2)	0.008	**0.584**	0.090	-0.034	0.865
Loss of meaning/purpose in life (F2)	-0.005	**0.828**	0.125	0.112	0.887
Loneliness (F3)	-0086	0.385	**0.682**	-0.013	0.897
Others judging me (F3)	0.132	-0.326	**0.780**	0.135	0.837
Fear from upcoming procedure (F1)	**0.925**	0.001	0.144	-0.289	0.631
Fear from death (F1)	**0.857**	-0.072	-0.013	0.334	0.745
Fear from the unknown about diagnosis/treatment (F1)	**0.920**	0.136	-0.087	-0.117	0.603
Loss bodily function (F2)	0.090	**0.860**	-0.076	-0.070	0.868
Missing important events (F2)	0.134	**0.610**	0.049	0.265	0.907
God abandoned/punished me (F4)	-0.041	0.004	-0.027	**0.865**	0.803
Questioning my faith (F4)	-0.082	0.072	0.061	**0.828**	0.785
Concerns about the afterlife (F4)	0.476	0.065	-0.082	**0.562**	0.875
Inadequate support from family (F3)	-0.033	0.031	**0.908**	0.048	0.864
Marital troubles (F3)	0.104	-0.050	**0.529**	0.221	0.877
Percentage of variance explained	41.81	16.05	10.48	6.97	**-**
Cronbach’s alpha	0.976	0.982	0.978	0.961	**-**

KMO = 0.844; Bartlett’s statistic = 5113.7 (df = 153; p < 0.001); total variance explained = 75.31%

### Bivariate analysis taking anxiety and depression as dependent variables

Higher anxiety was significantly associated with increased depression, all stressor groups (i.e., illness apprehension, hopelessness, social isolation, and spiritual concerns), higher financial burden, and higher household crowding index (i.e., lower socio-economic status). It was also associated with lower perceived social support and spiritual well-being. Higher depression was significantly associated with the same variables as anxiety, except the household crowding index (no significant association found) (Table 4). In addition, higher mean anxiety/depression scores were found in widowed participants compared to the other categories, in those with a primary level of education compared to the other categories, in those who do not have private insurance, in those who were admitted in a two-bed room compared to one bed, and in those who have cardiovascular problems, diabetes, neurological and psychiatric diseases compared to not. Moreover, higher anxiety scores were significantly found in patients who have hypertension compared to not (Table 5).

**Table 4:** Correlation of Continuous Variables with Anxiety and Depression

	A	D	PSS	SP	SF1	SF2	SF3	SF4	Age	FB	HCI	
Anxiety (A)	1											
Depression (D)	0.86***	1										
Perceived Social support (PSS)	− 0.50***	− 0.47***	1									
Spiritual well-being (SP)	− 0.80***	− 0.76***	0.58***	1								
Stressors Factor 1 (SF1) : Illness Apprehension	0.15**	0.11*	− 0.03	− 0.07	1							
Stressors Factor 2 (SF2): Hopelessness	0.78***	0.72***	− 0.42***	− 0.70***	0.06	1						
Stressors Factor 3 (SF3): Social Isolation	0.53***	0.46***	− 0.67***	− 0.58***	0.04	0.48***	1					
Stressors Factor 4 (SF4): Spiritual Concerns	0.53***	0.53***	− 0.41***	− 0.67***	0.11*	0.45***	0.40***	1				
Age	0.06	0.09	0.04	− 0.03	− 0.08	0.10*	− 0.16**	− 0.12*	1			
Financial burden (FB)	0.30***	0.19***	− 0.17***	− 0.22***	0.03	0.22***	0.22***	0.10*	− 0.02	1		
Household crowding index (HCI)	0.15**	0.05	− 0.03	− 0.04	0.05	0.06	0.04	0.02	− 0.30***	0.32***	1	

***p < 0.001; **p < 0.01; *p < 0.05

**Table 5:** Bivariate Analysis of Factors Associated with Anxiety and Depression

Variable	Anxiety (mean ± SD)	P	Depression (mean ± SD)	P
**Gender**		0.286		0.343
Male	20.23 ± 7.94		9.30 ± 4.91	
Female	21.05 ± 8.34		9.76 ± 5.37	
**Marital Status**		**< 0.001**		**< 0.001**
Single	18.74 ± 8.47		8.64 ± 5.09	
Married	21.12 ± 8.16		9.84 ± 5.29	
Divorced	19.47 ± 6.83		8.53 ± 4.37	
Widowed	25.89 ± 7.20		12.43 ± 4.82	
**Education**		**< 0.001**		**< 0.001**
Primary	24.63 ± 8.96		11.10 ± 5.66	
Complementary	22.17 ± 7.86		10.46 ± 5.09	
Secondary	20.90 ± 7.98		9.66 ± 4.94	
University	17.87 ± 7.42		8.17 ± 4.97	
**Private Insurance**		**< 0.001**		**< 0.001**
No	22.56 ± 8.07		10.51 ± 5.18	
Yes	17.71 ± 7.39		8.06 ± 4.76	
**National Social Security (NSS)**		0.063		**0.032**
No	21.28 ± 8.17		10.00 ± 5.18	
Yes	19.84 ± 8.10		8.95 ± 5.07	
**Lebanese Army**		0.112		0.527
No	20.31 ± 8.42		9.47 ± 5.39	
Yes	21.71 ± 7.27		9.78 ± 4.41	
**Ministry of Public Health (MOPH)**		**< 0.001**		**< 0.001**
No	20.08 ± 7.95		9.18 ± 4.98	
Yes	26.23 ± 8.14		13.02 ± 5.52	
**Number of Beds in the Room**		**< 0.001**		**< 0.001**
One bed	15.08 ± 6.96		7.43 ± 5.28	
Two beds	21.60 ± 7.97		9.90 ± 5.05	
**Comorbidities**				
Cardiovascular		**0.011**		**0.027**
No	20.02 ± 8.33		9.19 ± 5.26	
Yes	22.14 ± 7.57		10.36 ± 4.84	
Hypertension		**0.011**		0.153
No	19.99 ± 8.26		9.31 ± 5.18	
Yes	22.07 ± 7.77		10.05 ± 5.09	
Diabetes		**0.038**		**0.029**
No	20.29 ± 8.22		9.30 ± 5.03	
Yes	22.35 ± 7.71		10.67 ± 5.56	
Chronic kidney disease		0.207		0.087
No	20.50 ± 8.10		9.38 ± 4.97	
Yes	22.14 ± 8.60		11.14 ± 6.50	
Neurological		**< 0.001**		**< 0.001**
No	19.57 ± 8.00		9.02 ± 5.12	
Yes	24.37 ± 7.60		11.32 ± 4.88	
Psychiatric		**< 0.001**		**< 0.001**
No	19.92 ± 7.81		9.14 ± 5.01	
Yes	31.45 ± 4.91		15.41 ± 3.46	

Numbers in bold indicate significant p-values

### Multivariable analysis taking anxiety and depression as dependent variables

The results of the multivariable analysis, taking anxiety as the dependent variable, showed that higher spiritual well-being (Beta=-0.43) was significantly associated with lower anxiety, whereas increased illness apprehension (Beta = 0.69) and hopelessness (Beta = 1.37), being married (Beta = 1.17) or divorced (Beta = 1.38) compared to single, being admitted to a two-bed room compared to one bed (Beta = 1.59), higher financial burden (Beta = 0.24), and higher household crowding index (i.e., lower socio-economic status) (Beta = 1.60) were significantly associated with higher anxiety (Table 6, Model 1).

The results of the multivariable analysis, taking depression as the dependent variable, showed that higher spiritual well-being (Beta=-0.29) was significantly associated with lower depression, whereas increased hopelessness (Beta = 0.82) and being married (Beta = 0.79) compared to single were significantly associated with higher depression (Table 6, Model 2).

**Table 6:** Multivariable Analyses

Variable	Unstandardized Beta	Standardized Beta	*p*	95% CI	VIF
**Model 1: Linear regression (using the ENTER model) taking the anxiety score as the dependent variable (Nagelkerke R** ^2^ ** = 77.2%)**					
Perceived Social support	-0.02	-0.04	0.242	-0.05; 0.01	2.17
Spiritual Well-Being	-0.43	-0.44	**< 0.001**	-0.52; -0.35	3.60
Illness Apprehension	0.69	0.09	**< 0.001**	0.34; 1.04	1.07
Hopelessness	1.37	0.37	**< 0.001**	1.13; 1.62	2.20
Social Isolation	0.18	0.04	0.319	-0.18; 0.55	2.41
Spiritual Concerns	0.01	0.001	0.971	-0.58; 0.60	1.94
Married vs. single*	1.17	0.07	**0.019**	0.20; 2.15	1.76
Divorced vs. single*	1.38	0.06	**0.046**	0.03; 2.73	1.81
Widowed vs. single*	1.06	0.04	0.198	-0.55; 2.67	1.45
Complementary education vs. primary*	-0.05	-0.003	0.941	-1.43; 1.32	2.63
Secondary education vs. primary*	-0.31	-0.02	0.654	-1.67; 1.05	3.03
University education vs. primary*	-0.24	-0.01	0.751	-1.69; 1.22	3.38
Financial burden	0.24	0.06	**0.037**	0.01; 0.46	1.50
Household crowding index	1.60	0.08	**0.003**	0.53; 2.66	1.25
Private insurance (yes vs. no*)	0.24	0.01	0.619	-0.71; 1.19	1.59
MOPH (yes vs. no*)	0.38	0.01	0.576	-0.96; 1.71	1.14
Number of beds in the room (two vs. one*)	1.59	0.07	**0.011**	0.37; 2.81	1.35
Neurological comorbidities (yes vs. no*)	-0.01	-0.001	0.992	-1.00; 0.99	1.292
Psychiatric comorbidities (yes vs. no*)	1.56	0.05	0.078	-0.18; 3.29	1.34
**Model 2: Linear regression (using the ENTER model) taking the depression score as the dependent variable (Nagelkerke R**^**2**^ **= 65.9%)**					
Perceived Social support	-0.02	-0.07	0.110	-0.05; 0.01	2.17
Spiritual Well-Being	-0.29	-0.46	**< 0.001**	-0.35; -0.22	3.59
Illness Apprehension	0.18	0.04	0.200	-0.09; 0.45	1.07
Hopelessness	0.82	0.35	**< 0.001**	0.63; 1.01	2.20
Social Isolation	-0.13	-0.04	0.370	-0.41; 0.15	2.40
Spiritual Concerns	0.24	0.04	0.307	-0.22; 0.69	1.94
Married vs. single*	0.79	0.08	**0.039**	0.04; 1.55	1.75
Divorced vs. single*	0.41	0.03	0.441	-0.63; 1.44	1.76
Widowed vs. single*	0.25	0.01	0.690	-0.98; 1.48	1.41
Complementary education vs. primary*	0.82	0.07	0.127	-0.24; 1.88	2.60
Secondary education vs. primary*	0.76	0.07	0.158	-0.30; 1.81	3.03
University education vs. primary*	0.83	0.07	0.149	-0.30; 1.95	3.36
Financial burden	0.02	0.01	0.807	-0.14; 0.19	1.40
Private insurance (yes vs. no*)	-0.14	-0.01	0.703	-0.87; 0.59	1.58
MOPH (yes vs. no*)	0.71	0.04	0.179	-0.32; 1.74	1.13
Number of beds in the room (two vs. one*)	-0.35	-0.02	0.469	-1.29; 0.60	1.35
Neurological comorbidities (yes vs. no*)	-0.21	-0.02	0.589	-0.97; 0.55	1.27
Psychiatric comorbidities (yes vs. no*)	0.55	0.03	0.416	-0.78; 1.89	1.33

*Reference group

### Moderators between stressors and depression/anxiety

**Fig. 1:**
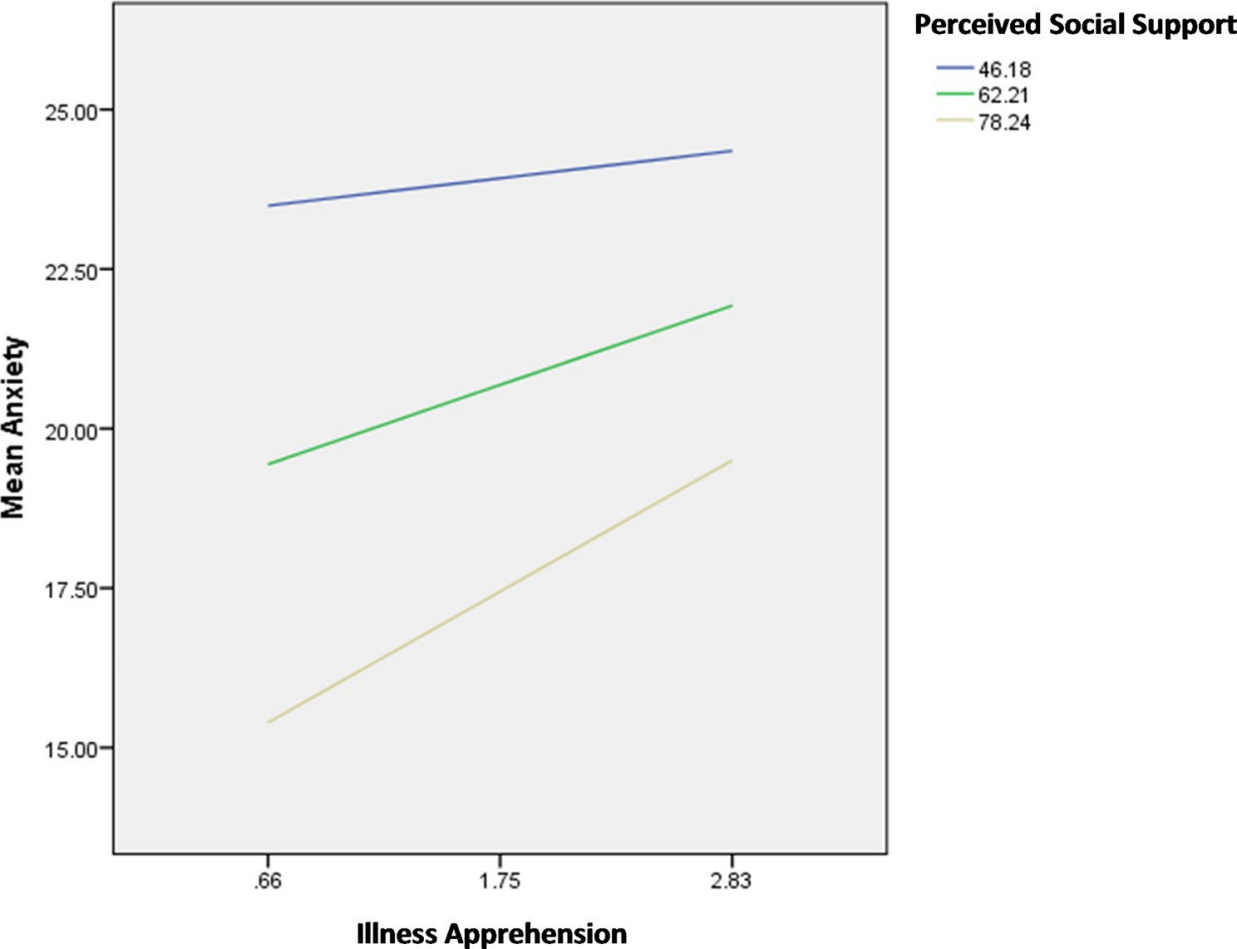
Interaction illness apprehension by perceived social support on anxiety

**Fig. 2:**
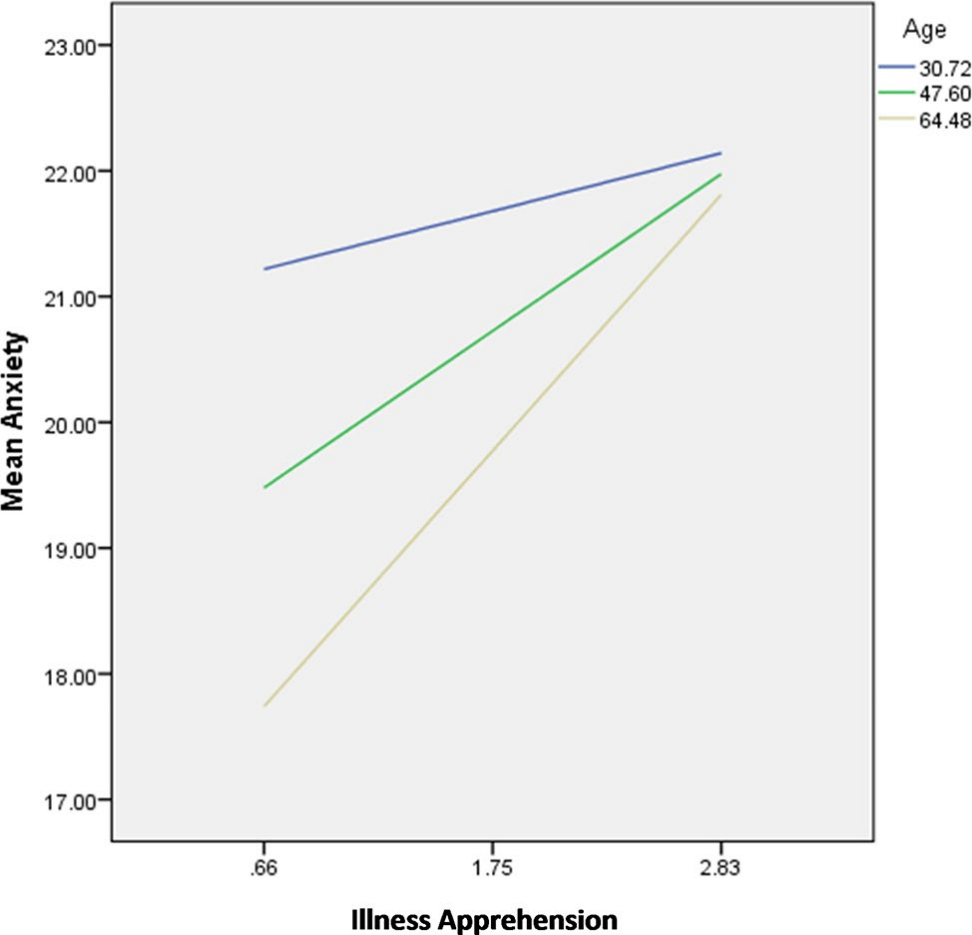
Interaction illness apprehension by age on anxiety

The results of the moderation analysis are summarized in Table 7. The interactions illness apprehension by perceived social support/age were significantly associated with anxiety; in patients with high illness apprehension, having high perceived social support (Fig. 1) and older age (Fig. 2) were significantly associated with lower anxiety.

**Table 7:** Moderation Analysis

	Anxiety			Depression		
	Beta	*p*	95% CI	Beta	*p*	95% CI
**Illness Apprehension**						
Perceived Social support	0.05	0.004	0.02; 0.08*	0.01	0.258	-0.01; 0.03
Spiritual well-being	0.03	0.176	-0.01; 0.08	-0.03	0.062	-0.06; 0.002
Age	0.04	0.013	0.01; 0.08*	0.03	0.008	0.01; 0.05*
Gender	0.78	0.209	-0.44; 1.99	0.23	0.581	-0.59; 1.04
Household crowding index	-1.43	0.067	-2.96; 0.10	-1.00	0.056	-2.03; 0.03
Number of comorbidities	0.10	0.663	-0.36; 0.57	0.04	0.799	-0.27; 0.35
**Hopelessness**						
Perceived Social support	-0.002	0.777	-0.02; 0.01	0.001	0.783	-0.01; 0.01
Spiritual well-being	-0.02	0.220	-0.04; 0.01	-0.03	0.007	-0.04; -0.01*
Age	0.001	0.834	0.01; 0.01	0.002	0.683	-0.01; 0.01
Gender	-0.08	0.698	-0.51; 0.34	0.06	0.686	-0.24; 0.37
Household crowding index	-0.20	0.468	-0.75; 0.34	-0.42	0.034	-0.82; -0.03*
Number of comorbidities	0.06	0.495	-0.12; 0.25	0.06	0.393	-0.07; 0.19
**Social Isolation**						
Perceived Social support	0.02	0.197	-0.01; 0.04	0.01	0.086	-0.002; 0.03
Spiritual well-being	0.05	0.005	0.02; 0.09*	0.03	0.063	-0.001; 0.05
Age	-0.01	0.598	-0.03; 0.02	0.003	0.743	-0.01; 0.02
Gender	0.24	0.524	-0.50; 0.98	0.18	0.492	-0.34; 0.70
Household crowding index	-0.28	0.544	-1.18; 0.62	-0.43	0.175	-1.06; 0.19
Number of comorbidities	0.07	0.648	-0.24; 0.38	0.14	0.207	-0.08; 0.35
**Spiritual Concerns**						
Perceived Social support	0.07	< 0.001	0.04; 0.11*	0.03	0.009	0.01; 0.06*
Spiritual well-being	0.11	0.002	0.04; 0.19*	0.01	0.595	-0.04; 0.07
Age	0.01	0.589	-0.03; 0.05	0.03	0.028	0.003; 0.05*
Gender	-0.82	0.237	-2.17; 0.54	-0.34	0.455	-1.25; 0.56
Household crowding index	-1.31	0.106	-2.90; 0.28	-1.51	0.005	-2.57; -0.45*
Number of comorbidities	-0.48	0.097	-1.04; 0.09	-0.02	0.901	-0.40; 0.35

*indicates significant moderation. Models were adjusted over the following variables: marital status, education, insurance type, financial burden, and number of beds in the room

**Fig. 3:**
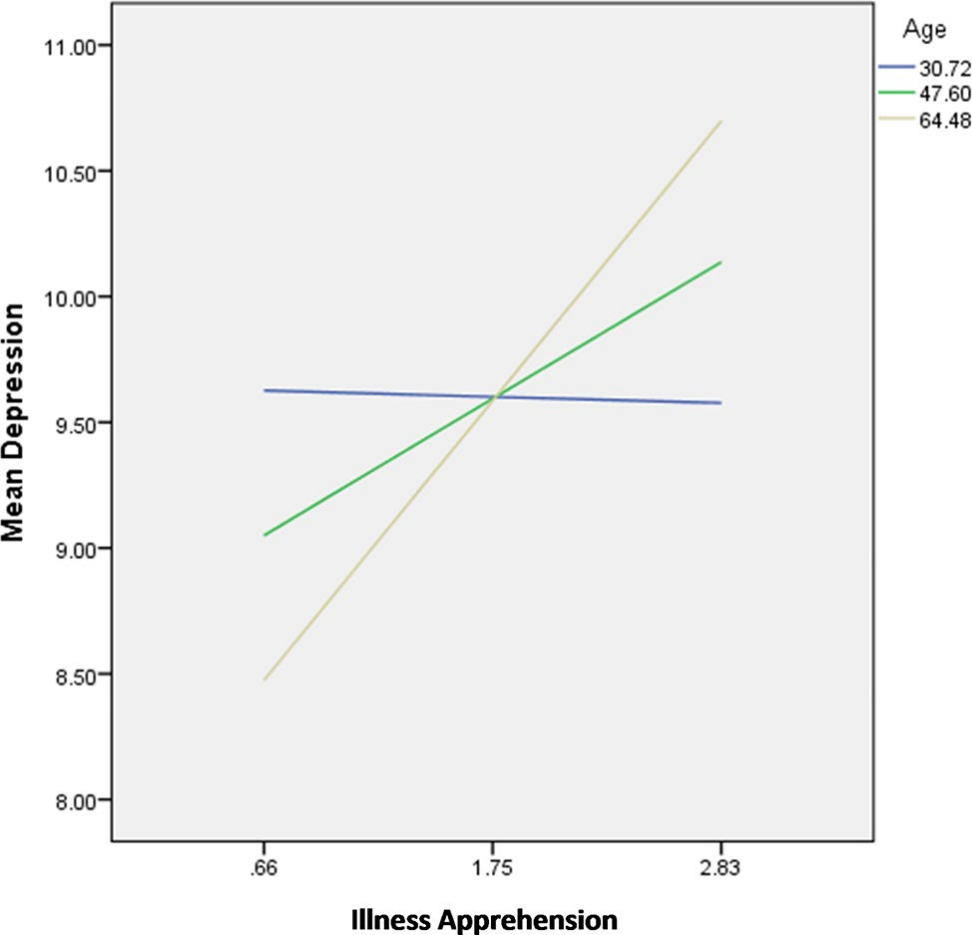
Interaction illness apprehension by age on depression

The interactions illness apprehension by age were significantly associated with depression; in patients with high illness apprehension, younger age (Fig. 3) was significantly associated with lower depression.

**Fig. 4:**
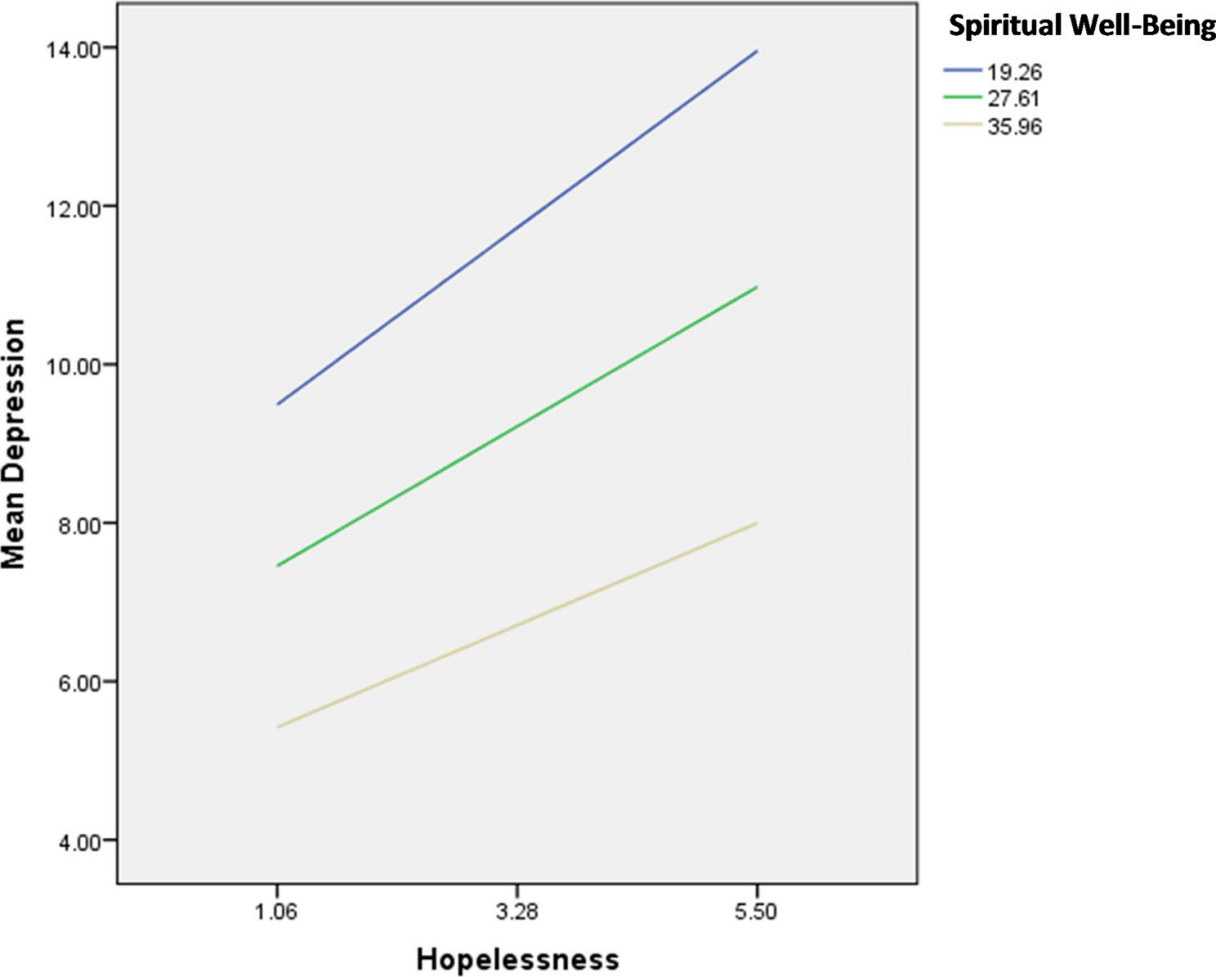
Interaction hopelessness by spiritual well-being on depression

**Fig. 5:**
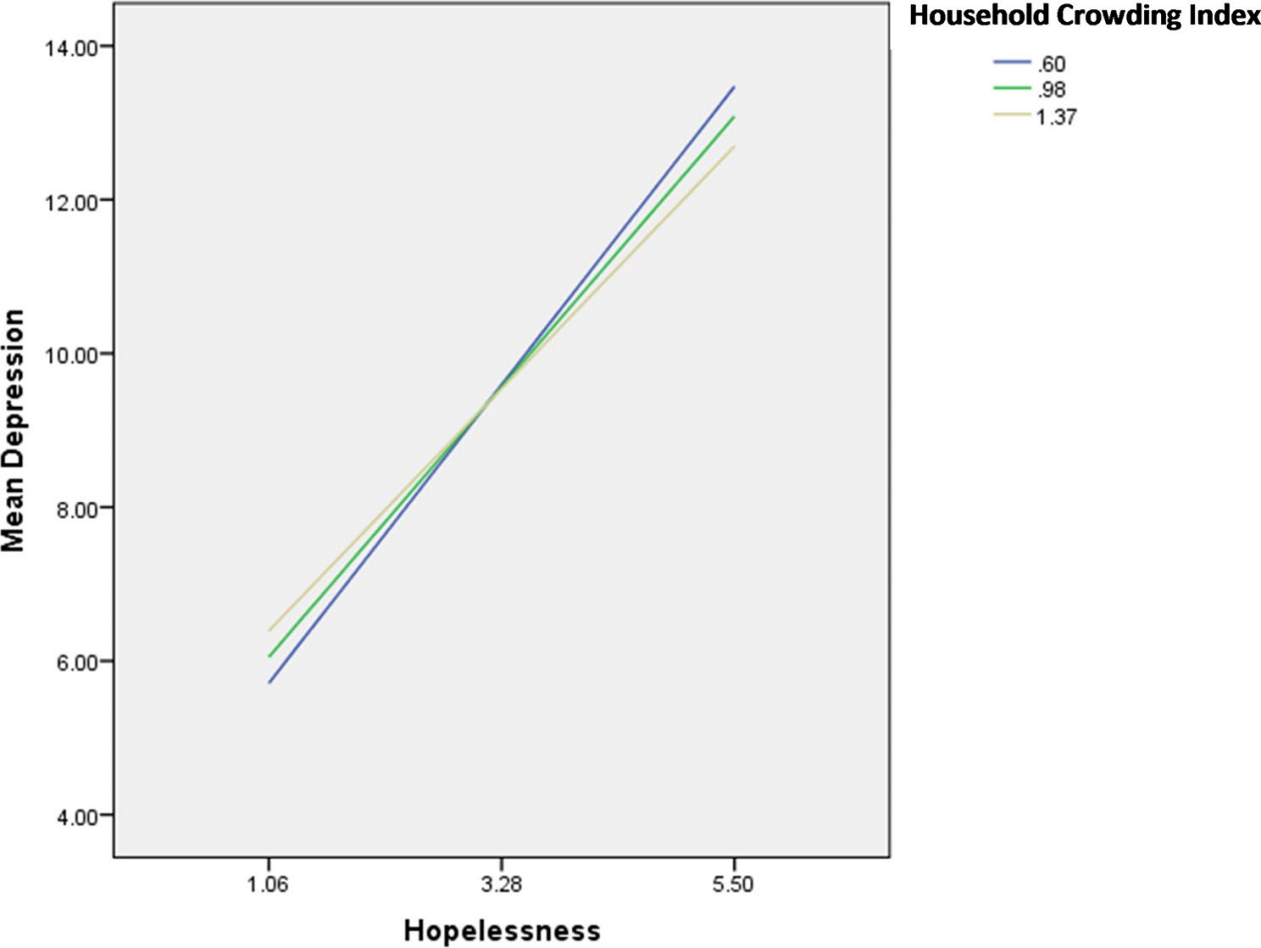
Interaction hopelessness by household crowding index on depression

The interactions hopelessness by spiritual wellbeing/household crowding index were significantly associated with depression; in patients with high hopelessness, having high spiritual wellbeing (Fig. 4) and a high household crowding index (lower socioeconomic status) (Fig. 5) were significantly associated with lower depression.

**Fig. 6:**
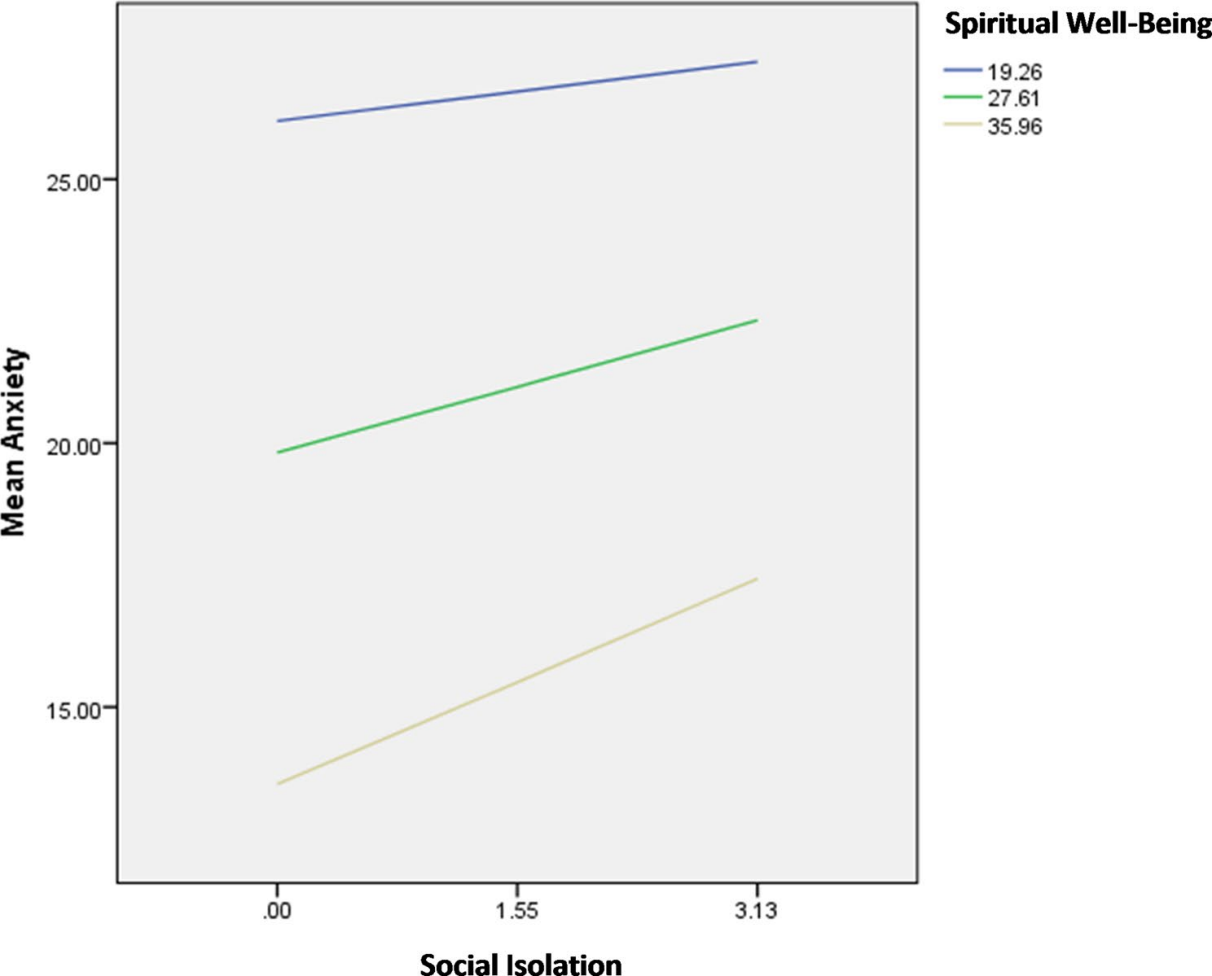
Interaction social isolation by spiritual well-being on anxiety

The interaction social isolation by spiritual wellbeing was significantly associated with anxiety; in patients with high social isolation, having high spiritual wellbeing was significantly associated with lower anxiety (Fig. 6).

**Fig. 7:**
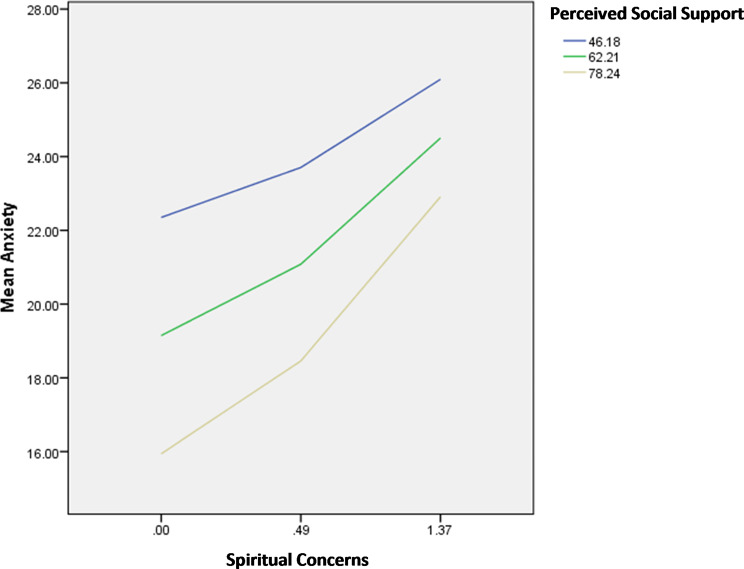
Interaction spiritual concerns by perceived social support on anxiety

**Fig. 8:**
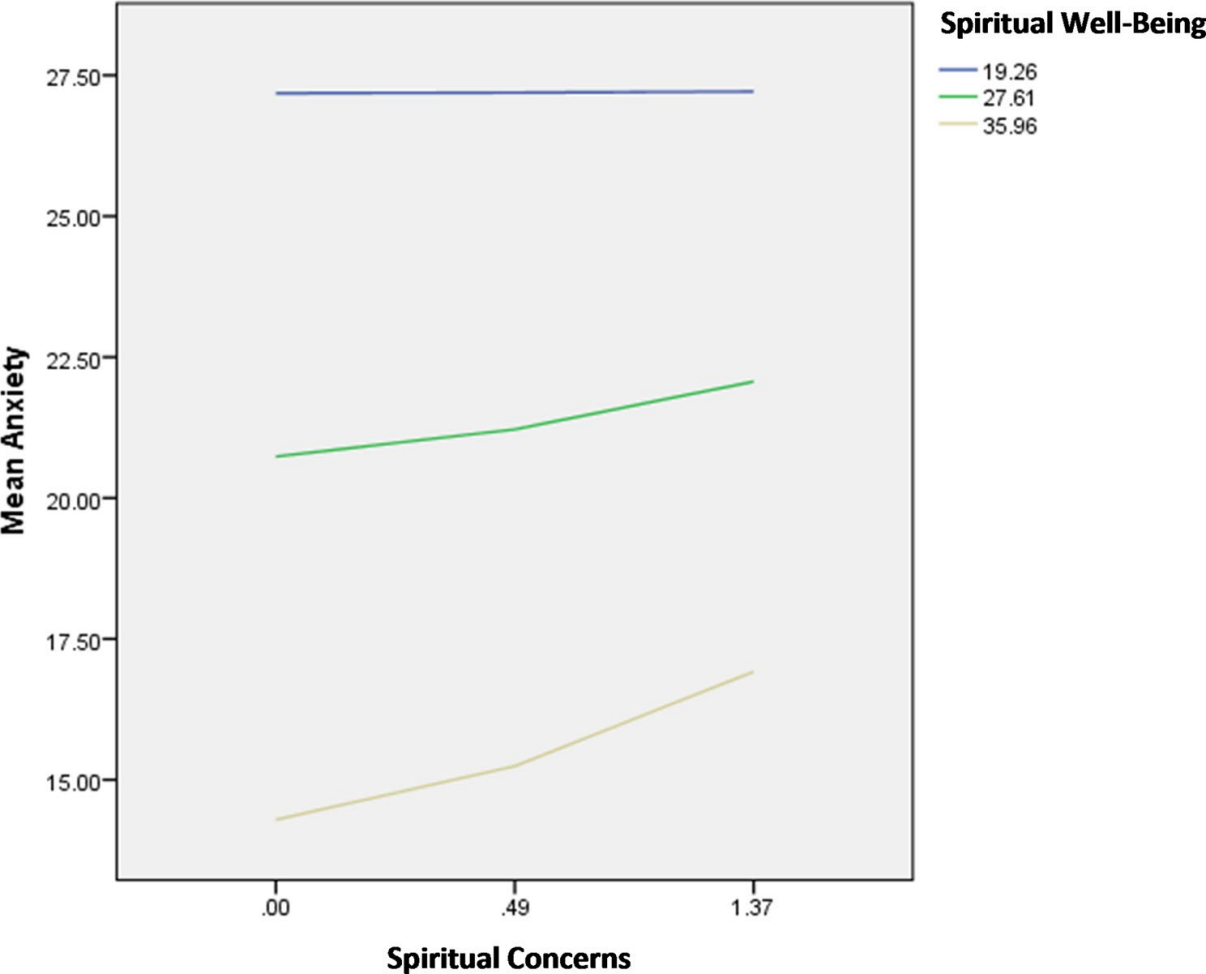
Interaction spiritual concerns by spiritual well-being on anxiety

The interactions spiritual concerns by perceived social support/spiritual wellbeing were significantly associated with anxiety; in patients with high spiritual concerns, having high perceived social support (Fig. 7) and high spiritual well-being (Fig. 8) were significantly associated with lower anxiety.

**Fig. 9:**
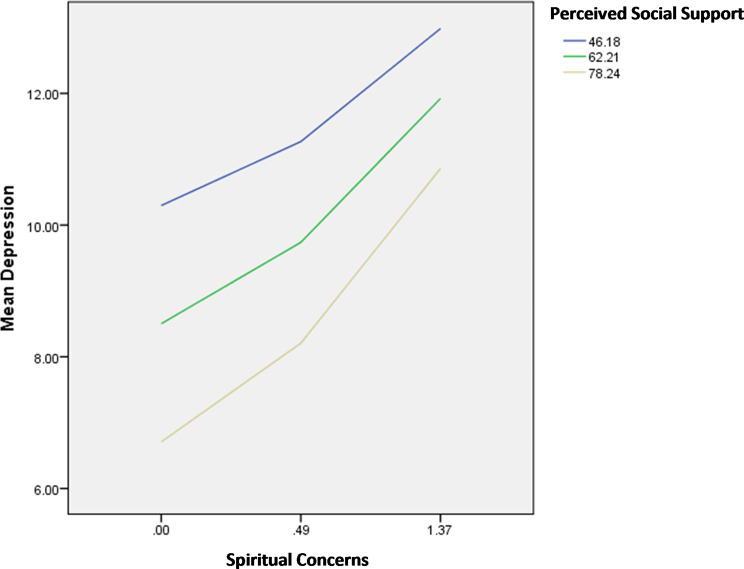
Interaction spiritual concerns by perceived social support on depression

**Fig. 10:**
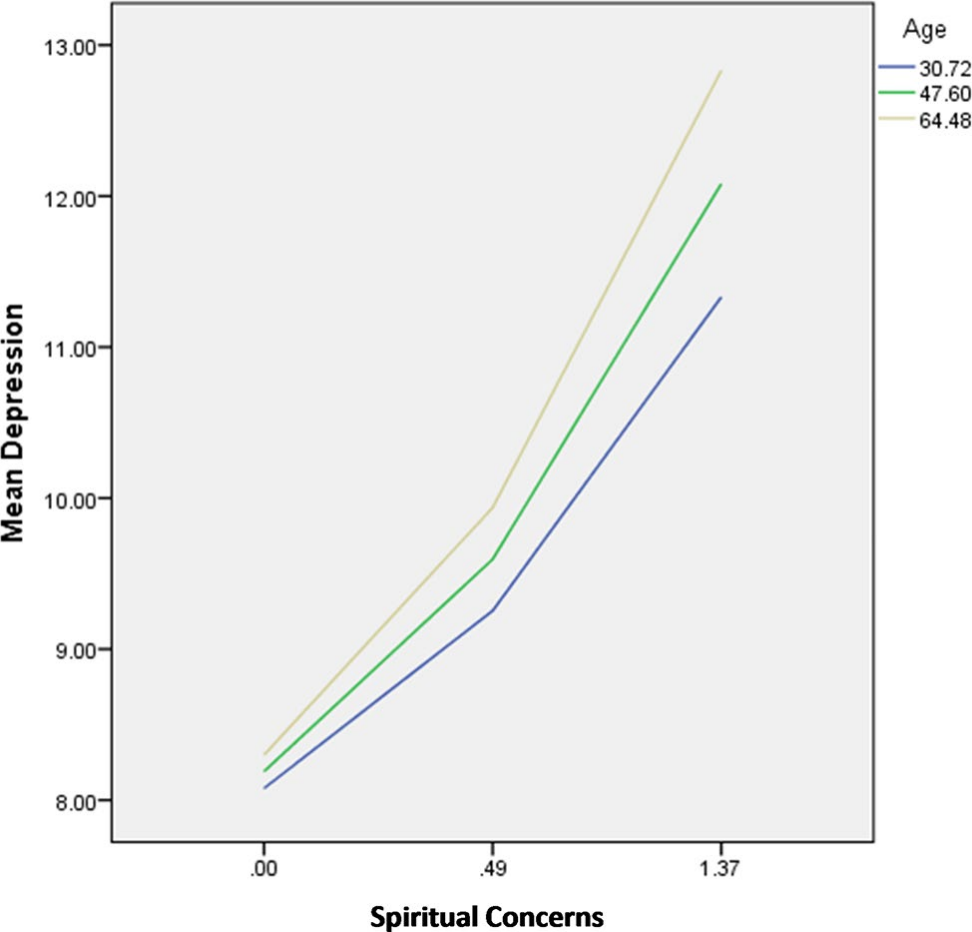
Interaction spiritual concerns by age on depression

**Fig. 11:**
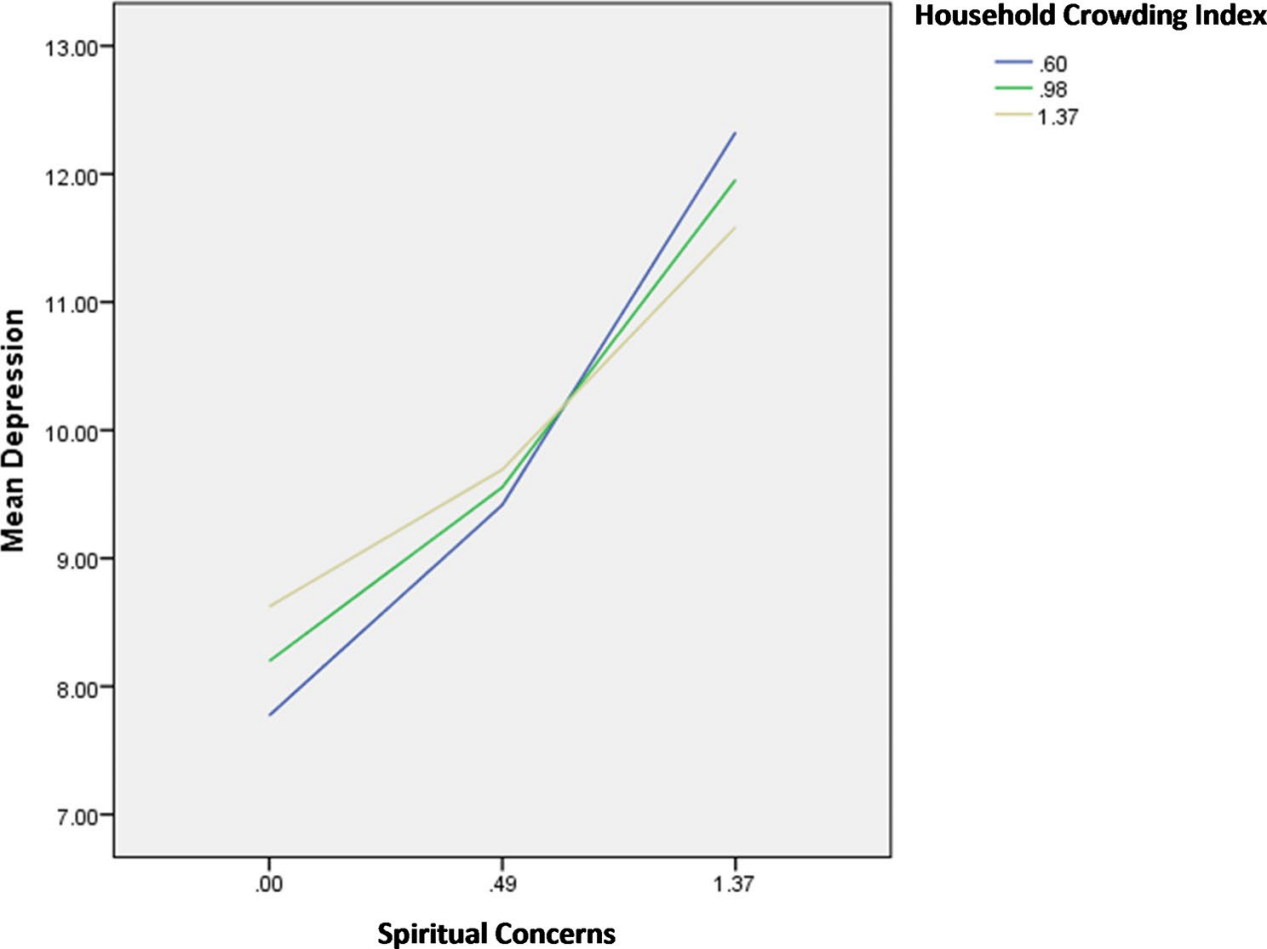
Interaction spiritual concerns by household crowding index on depression

The interactions spiritual concerns by perceived social support/age/household crowding index were significantly associated with depression; in patients with high spiritual concerns, having high perceived social support (Fig. 9), younger age (Fig. 10) and high household crowding index (lower socioeconomic status) (Fig. 11) were significantly associated with lower depression.

## Discussion

Within this study, pain was the most endorsed stressor by the participants, in line with several previous studies reporting a high prevalence of pain in hospitalized patients [21, 49, 50]. The factor analysis identified four multi-item factors measuring stressors among our hospitalized sample, which were related to the following themes: illness apprehension/fear, hopelessness/usefulness, social isolation, and spiritual concerns. In fact, illness-related fear and uncertainty in illness (i.e., illness apprehension) are well-established concerns in the healthcare setting, which may also exert detrimental impacts on patients’ mental health and quality of life [8, 21, 51]. Further, in line with our results, a meta-analysis found that lung cancer patients expressed illness-associated emotional experiences related to fear, worries, uncertainties, despair, uselessness, dependency, and loneliness [52]. In addition, being away from family members (i.e., social isolation) has also been identified as a potent source of post-surgical anxiety [9]. Our findings are also consistent with prior research indicating high levels of depression/anxiety among hospitalized patients who had the need to speak to a spiritual advisor, feel more supported by their relatives, and feel less abandoned [53]. Actually, spiritual distress and suffering related to lack of meaning in life (i.e., hopelessness and spiritual concerns) are common struggles identified in medical disease patients [21], such as cancer patients undergoing chemotherapy [54].

Furthermore, our study showed that patients who felt hopeless and useless during hospitalization had higher symptoms of depression and anxiety. Similarly to our results, McKenzie et al. showed that pessimism and worthlessness were highly correlated with major depression among hospitalized, medically ill patients [55]. Additionally, in line with previous research [21, 51, 56], patients with high illness apprehension displayed a significantly greater level of anxiety. Our study thus extends the results of prior research conducted among general and specific subgroups of inpatients, suggesting their relevance among all Lebanese inpatients regardless of disease type and severity.

On another hand, our results indicated that being married/divorced compared to single was significantly associated with higher scores of anxiety. In addition, married participants had higher levels of depression compared to single inpatients, in line with prior research among patients diagnosed with oral cancer [57]. Our findings thus speculate that single patients might be less prone to depressive/anxiety symptoms in the hospital context since they do not have additional responsibilities to a partner or child/children. Moreover, patients with lower socio-economic status and higher financial burden exhibited higher anxiety symptoms in our population. Similarly, low income and being unemployed were found to be among the prime factors associated with psychological distress among hospitalized patients with diabetes mellitus in Saudi Arabic and cancer patients attending follow-up in Ethiopia, respectively [16, 58]. Certainly, patients who find it difficult to afford the costs of their treatment and hospitalization would be more susceptible to anxiety, fearing the progress of their disease if they could not obtain the necessary care. In line with this perspective, patients residing in a two-bed room compared to one-bed were more vulnerable to anxiety. The latter association may also be related to the socio-economic status of the patients who cannot afford a private hospital room. From another standpoint, those patients’ anxiety might result from hearing the burdens and complaints of their hospital roommates or from witnessing a bad course of their disease.

In contrast, our findings revealed that greater spiritual well-being was significantly associated with lower depression and anxiety symptoms. Moreover, in patients experiencing high levels of stressors, those with high spiritual well-being and perceived social support had less depressive/anxiety symptoms. Consistent with our findings, a previous study showed that patients with serious and advanced diseases who had greater spiritual well-being as measured by the FACIT-Sp; including beliefs about the role of faith in illness and meaning, peace, and purpose in life; were considerably less depressed and anxious [59]. Another study observed that perceived social support was related to a better quality of life in oral cancer patients in China [60]. These observations may have paramount clinical implications for implementing targeted interventions (i.e., enhancing social support and promoting spiritual well-being) to alleviate in-hospital depression and anxiety across all medical units.

Concerning the moderating roles of sociodemographic characteristics, a lower socioeconomic status had a negative moderating effect in the association between stressors (hopelessness and spiritual concerns) and depression. This finding implies that people from lower socioeconomic backgrounds may face more difficulties and thus develop coping strategies and resilience to stress [61, 62], which may contribute to a lower susceptibility to depressive symptoms in response to emotional stressors the hospital. Finally, our study found that the associations between stressors and depression were weaker in younger people, whereas older people had lower anxiety in response to stressors. In line with our results, a prospective longitudinal study on cancer patients found that anxiety declines with age while depression rises [30]. Another study also demonstrated that older patients had more depressive symptoms post-stroke [63]. In light of our findings, we cautiously suggest that programs to reduce anxiety in younger patients are required, while more resources and attention should be directed towards identifying and treating depression in older hospitalized patients.

### Clinical implications

This study is important for broadening physicians’ understanding of a wide range of stressors experienced by general hospitalized patients, as many of which may be present at subclinical levels and hence go unnoticed for effective psychological/psychiatric assessment and management. Understanding the factors that are favorably and adversely linked with their depressed and anxiety symptoms, in order to create efficient screening programs and clinical preventive/therapeutic interventions, could help to improve the hospital experience for all patients. Precisely, our study prompts hospital-wide interventions in Lebanon, who would work towards developing strong social support networks for inpatients (e.g., extending hospitals’ visiting hours, encouraging patients’ communication with their family/entourage, family counseling, etc.). Further, our results provided a tool to identify inpatients who need support, and highlighted the need of promoting their spiritual well-being; hence, they might benefit the hospital personnel (i.e., hospital chaplains) who operate through all hospital units.

### Limitations

Despite its important contribution to the literature tackling the psychological well-being of Lebanese hospitalized patients, the current study is not devoid of certain limitations. First, its cross-sectional nature allows to only capture a snapshot in time, hence hindering the ability to establish causal and temporal associations. Second, the symptoms were self-reported; although anonymous self-report surveys limit social undesirability, patients might misunderstand some statements. Third, we recruited the patients from a single hospital, which limits the generalizability of our results to the whole Lebanese population. Fourth, by studying moderators, this study provided a deeper comprehensive analytical approach to the relationships between stressors in the hospital and mental health outcomes; nevertheless, we found a very high negative correlation between anxiety and spiritual well-being in our sample (i.e., -0.8), which means that higher spiritual well-being was very closely linked to lower anxiety in our population. This high correlation might have also significantly influenced the moderation analysis. Finally, not all possible confounding factors were examined in the present study. For instance, our study did not consider the time of stay in the hospital, the hospital admission frequency, or the medications used as independent variables, which might have affected the outcomes. Further research with a wider range of predictors are highly encouraged to complement our results.

## Conclusion

Our study characterized the principal stressors encountered during hospitalization, underscoring their associations with Lebanese inpatients’ mental health. On the other hand, as perceived social support and spiritual well-being acted as negative moderators of these associations, intervention programs aimed at enhancing such adaptive coping techniques are strongly called upon to palliate the psychological distress of patients in hospital settings. Future prospective studies are needed to deepen our insights into the variety of biopsychosocial factors eliciting distress across the wide hospital inpatient population, and their mitigating counter-force, in order to enact preventive/therapeutic interventions that would benefit a large number of patients.

## Data Availability

The datasets generated and/or analyzed during the current study are not publicly available due to restrictions from the ethics committee, but are available from the corresponding author on reasonable request.
